# Supplementary material for: Effect of chicken manure and chemical fertilizer on the yield and qualities of white mugwort at dissimilar harvesting times
Source: PLoS One. 2022 Apr 26;17(4):e0266190. doi: 10.1371/journal.pone.0266190 (PMC9041803; doi:10.1371/journal.pone.0266190)
Supplement: Attachment — Submitted filename: Response to Reviewers.docx [file pone.0266190.s001.docx]

**Review’s Comment & Response to Reviewer PLOS ONE: PONE-D-21-36764**

| **Reviewer** | **Reviewer’s Comment** | **Respond to Reviewer** |
| --- | --- | --- |
| **1** | **Overall comments:**  1. The author needs to improve the English language and writing style of this article.  2. Data information: The author must arrange the data in both table and graph. Actually, data on chlorophyll content, total phenolic content, nitrate reductase activity, and DPPH radical scavenging capacity need to present in a graph with a standard error bar.  3. Part of Introduction:  The author needs to add more examples of the application of organic and chemical fertilizers for the cultivation of white mugwort plants, not for other vegetables crops or plants.  4. If the author would arrange a treatment with the combination of both chemical and organic fertilizers this article could be more reasonable and scientifically sound. | Thank you very much for your advice. We already asked a British English native-reader to revise our manuscript for improving the English language and writing style of this article as shown below;  **Title:**  Page 1: Line 1-2;  **From** “Effect of chicken manure and chemical fertilizer on the yield and qualities of white mugwort at different harvesting times”  **To** “Effect of chicken manure and chemical fertilizer on the yield and qualities of white mugwort at dissimilar harvesting times”  **Abstract:**  Page 2: Line 38-39;  **From** “The application of chicken manure at 10.08 t/ha produced the highest fresh weight at all harvest times.”  **To** “Chicken manure application at 10.08 t ha^-1^ produced the highest fresh weight at all times.”  **Introduction:**  Page 2: Line 48-49;  **From** “White mugwort (*Artemisia lactiflora* Wall. ex DC.) is one species of genus *Artemisia* that belongs to the Compositae family. It primarily exists in southeast Asia.”  **To** “White mugwort (*Artemisia lactiflora* Wall. ex DC.) is one species of genus *Artemisia* that belongs to the Compositae family and primarily exists in southeast Asia.”  Page 3: Line 54-56;  **From** “In addition, it has been demonstrated that the leaves of white mugwort are rich in total phenolic, ascorbic acid, and some essential minerals, such as iron and calcium [3].”  **To** “In addition, it has been shown that the leaves of white mugwort are rich in total phenolic, ascorbic acid, and some essential minerals, such as iron and calcium [3].”  **Results and Discussion: Yield quality (nitrate accumulation and phytochemical compounds)**  Page 14: Line 276-277;  **From** “The plants significantly accumulated more nitrate in the above-ground parts as nitrogen level in fertilizer increased.”  **To** “As nitrogen level increased, the plants accumulated more nitrate in the above-ground parts.”  Page 16: Line 333-335;  **From** “Since, the average rainfall in our research area during the third harvesting cycle (350 mm) was higher than that of the first and second harvesting cycles, 230 and 250 mm, respectively [47].”  **To** “Since the average rainfall in our research area during the third harvesting cycle (350 mm) was higher than that of the first and second harvesting cycles, 230 and 250 mm, respectively [47].”  Page 17: Line 337-339;  **From** “Previous studies revealed a significant positive correlation between phenolic compounds and antioxidant activity [39].”  **To** “Previous studies have revealed a significant positive correlation between phenolic compounds and antioxidant activity [41].”  Thank you very much for your advice. Considering your advice, we have edited the data on chlorophyll (a+b) content, nitrate concentration, total phenolic content, and DPPH radical scavenging capacity to be a graph with a standard error bar as shown in attached figure files (Fig 1-Fig 4). We have changed the part of in-text citation from Table 6-9 to Fig 1-4 in Page 13: Line 261, Page 14: 275, Page 15: 295 and Page 16: 319, respectively. In addition, we added all the figure captions in the manuscript text in read order, immediately following the paragraph where the figure is first cited as shown in  Page 14: Line 265-269 (Fig 1 caption),  Page 15: Line 287-291 (Fig 2 caption),  Page 15: Line 306-310 (Fig 3 caption),  Page 17: Line 346-350 (Fig 4 caption),    We try to find more research focusing on the application of organic and chemical fertilizers for the cultivation of white mugwort plants, however, there was no further research on the white mugwort plant but there were several studies on *Artemisia annua* the same genus Artemisia as white mugwort. Therefore, we added more detail of this in the Introduction and rearranged the information, as shown in Page 3-4: Line 57-90.  Thank you so much for your advice. The reason we would like to present this article is to show the data comparing between the type and doses of chemical fertilizer and organic fertilizers “chicken manure” that affect the growth and yield quality of white mugwort. It appears that to obtain quality products and reduce the risk of nitrate accumulation, chicken manure is more suitable than chemical fertilizer, as it was seen in the experiment where chicken manure at 10.08 t ha^-1^ can replace the use of chemical fertilizer and also gave the highest of total fresh yield. This data could be used as the sample of organic agricultural management for white mugwort production. However, we will arrange a treatment with the combination of both chemical and organic fertilizers in the further experiment for white mugwort production. |
|  | Specific Comments:  1. Total Plagiarism percentage is 36 % with the yellow color level in Turnitin that is not acceptable to my knowledge. The author should reduce the plagiarism from 36% to 20% with a blue color index in Turnitin software. In line 28-32, In line 103-104, Line 147-150, Line 160, Line 164-177, Line 179-182, Line 246-248, Line 287-288, Line 325-327 plagiarism percentage is not acceptable. Please reduce it.  2. Line 116: What types of natural extract were applied in the plot to control pests and diseases?  3. Line 112-115: Please “The organic fertilizer treatments with chicken manure were applied at the time of 113 transplantation, whereas the chemical fertilizer treatments were applied with complete fertilizer 114 25-7-7 grade at the time of transplantation, and with urea 46-0-0 grade at 14 days before harvest.” Change into “The doses of organic fertilizers “chicken manure” were applied at the time of transplanting, whereas the doses of chemical fertilizers were applied with complete fertilizer 114 25-7-7 grade at the time of transplanting, and with urea 46-0-0 grade at 14 days before harvest.  4. Please provide the analyzed data in SPSS software with a PDF file for the reviewer’s understanding.  5.Other | Thank you so much for your advice. We have rearranged all the sentences that you mention for reducing the plagiarism and checked with “Turnitin software” as shown the result of plagiarism percentage is approximately 20%.  Note:  **From** Page 2: Line 28-32;  “Fertilization is a crucial factor that influences soil fertility, plant growth, and crop quality. This study evaluated the effect of organic and chemical fertilizer on soil chemical properties, yield, and quality of white mugwort grown in extremely acid (pH 4.10) sandy loam soil. The field experiment conducted in Prachin Buri province was arranged in a completely randomized block design, with four replications.” changed to the sentence as shown in the same place  **To** Page 2: Line 28-32; “One of the key components that affects soil productiveness, plant growth, and crop quality is fertilization. The effect of fertilizer, both organic and chemical, on the extremely acid (pH 4.10) sandy loam soil chemical properties, yield, and quality of white mugwort grown were evaluated in this study. The field experiment arranged in a randomized complete block design, with four replications was conducted in Prachin Buri province, Thailand.”  **From** Page 5: Line 103-104;  “The experiment was conducted in a randomized complete block design with four replications.”  **To** Page 5: Line 103-104;  “A randomized complete block design with four replications was used to perform the study.”  **From** Page 7: Line 147-150;  “An area of 0.28 cm^2^ of the mugwort leaf was punched out and soaked in 10 mL of 80% (v/v) acetone to extract the chlorophyll. The supernatant was measured at 645 and 663 nm for the determination of the total chlorophyll content by using a spectrophotometer. The chlorophyll content was expressed as the content per unit surface area.”  **To** Page 7: Line 147-151;  “To determine the total chlorophyll content, a 0.28 cm^2^ section was removed from the white mugwort leaf. Then, to extract the chlorophyll the section was soaked in 10 mL of 80% (v/v) acetone. A spectrophotometer was used to measure the supernatant at 645 and 663 nm to determine the total chlorophyl content and the content was expressed as content per unit surface area.”  **From** Page 8: Line 160;  “Total phenolic content and DPPH radical scavenging assay”  **To** Page 8: Line 161;  “Phytochemical compounds”  **From** Page 8: Line 164-177;  “The homogenized sample (1 g of shoot fresh weight) was weighed and then 10 mL of 70% (v/v) ethanol was added using the ratio 1:10 w/v and stirred at 150 rpm in a shaker at 4^o^ C for 150 min. The extract was then separated from the residue by filtration through Whatman No. 1 filter paper. For the analysis of total phenolic content, 0.3 mL of the extract was transferred into a test tube, then 2.5 mL of 2 M Folin-Ciocalteu reagent and 2.0 mL of 7.5% (w/v) sodium carbonate were added and mixed. The mixture was incubated at room temperature, in darkness for 150 min and measured at a wavelength of 765 nm by using a spectrophotometer. The standard calibration curve was plotted by using gallic acid. The total phenolic content was expressed as gallic acid equivalents in mg gallic acid per 1 g fresh weight. For the DPPH assay, a modified method of Wang et al [25] and Jin et al [26] was carried out. Succinctly, 0.1 mL DPPH solution (0.1 mM in 95% ethanol) was mixed with 0.1 mL of the extracted samples with different concentrations in the microplate. The samples were kept in a dark condition at room temperature and after 30 min, the absorbance was measured by using a micro-plate reader at 517 nm. Trolox was used as the standard and results were expressed as mg Trolox equivalent antioxidant capacity (TEAC) per g fresh weight.”  **To** Page 8: Line 164-178;  “After weighing the homogenized sample (1 g of shoot fresh weight) using the ratio 1:10 w/v 10 mL of 70% (v/v) ethanol was added and stirred in a shaker at 150 rpm and 4^o^ C for 150 min. The extracted was separated from the residue by filtrating through a Whatman No. 1 filter paper. To find out the total phenolic content analysis, the extract (0.3 mL) was transferred to a test tube, 2.5 mL of 2 M Folin-Ciocalteu reagent and 2.0 mL of 7.5% (w/v) sodium carbonate were added and mixed. Subsequently, the mixture was incubated in the dark, at room temperature, for 150 min, afterward a spectrophotometer was used at a wavelength of 765 nm to measure the purity. The standard calibration curve was plotted by using gallic acid and total phenolic content was expressed as gallic acid equivalents in mg gallic acid per 1 g fresh weight. A modified method of the Wang et al [27] and Jin et al [28] method was carried out for DPPH assay. Succinctly, 0.1 mL of the extracted samples with different concentrations in the microplate were mixed with 0.1 mL DPPH solution (0.1 mM in 95% ethanol). The samples were kept at room temperature, in the dark condition. After 30 min, a micro-plate reader at 517 nm was used to measure the absorbance. The standard used was Trolox and results were expressed as mg Trolox equivalent antioxidant capacity (TEAC) per g fresh weight.”  **From** Page 8: Line 179-Page 9: Line 182;  “Analysis of variance in a randomized complete block design was conducted by using IBM SPSS Statistics, Version 26.0 software (IBM Corp., Armonk, NY, USA). The differences among the mean values of treatment were compared by using Duncan’s multiple range tests, with significance determined at p < 0.05.”  **To** Page 8: Line 180- Page 9: Line 184;  “Analysis of variance in a randomized complete block design was conducted by using IBM SPSS Statistics, Version 26.0 software (IBM Corp., Armonk, NY, USA) was used to conduct analysis of variance in a randomized complete block design. The mean values of treatment difference were compared by using Duncan’s multiple range tests, with the significance determined at p < 0.05.”  **From** Page 12: Line 245-Page 13: Line 248;  “Iqbal et al [33] suggested that the application of high N concentration improved the N uptake used to form soluble nitrogenous compounds such as free amino acids like proline, which act as osmotic regulators and contributes to reducing the osmotic potential and thus maintains cell turgidity.”  **To** Page 12: Line 247- Page 13: Line 250;  “The application of high N concentration improves the N uptake in plant and is employed to form soluble nitrogenous compounds, for example free amino acids such as proline, which function as osmotic regulators and take part in reducing the osmotic potential and so maintain cell turgidity [35].”  **From** Page 15: Line 287-289;  “This may be attributed to the key substance for phenolic synthesis, such as phenylalanine, that preferentially flows into chain protein synthesis rather than toward the pathway of phenolic synthesis for increasing plant biomass in response to high nitrogen levels [37].”  **To** Page 15: Line 298-301;  “Being possibly accredited to the elements necessary for phenolic synthesis, for example phenylalanine, which flows preferentially into chain protein synthesis in preference to the phenolic synthesis pathway, so increasing plant biomass in response to high nitrogen levels [39].”  **From** Page 18: Line 325-327;  “However, the correlation between the phytochemical content and environmental conditions remains controversial. Future work should explore the correlation between these parameters in more detail.”  **To** Page 17: Line 341-344;  “Nevertheless, the phytochemical content to environmental conditions correlation continues to be contentious. Future work should investigate the correlation between the phytochemical content to environmental conditions in greater detail.”  The type of natural extracts are neem extracts. Therefore; I have revised the sentence at Line 116 as  Page 5: Line 116- Page 6: Line 117; “Neem extracts were sprayed to eradicate disease and insects when spreading and mechanical methods were utilized to remove the weeds.”  Thank you very much for your advice. Considering your advice, we already edited the sentence following your suggestion that was changed into Page 5: Line 112-114 “The doses of organic fertilizers “chicken manure” were applied at the time of transplanting, whereas the doses of chemical fertilizers were applied with complete fertilizer 25-7-7 grade at the time of transplanting, and with urea 46-0-0 grade at 14 days before harvest.”  We already provided the analyzed data in SPSS software with a PDF file as the attached file.  We apologize that there is a mistake in the SD value of the CF 187.50 kg ha^-1^ treatment in Table 4, which we would like to change from 0.01 to 0.10. (Page 12: Line 239) |
|  |  |  |
|  |  |  |
|  |  |  |
| **2** | Although the manuscript was written in standard English, statistical analysis were performed properly, I found the concept of this manuscript very common and weak particularly to be published in this journal. I believe authors can improve this manuscript by either improving the presentation and/or present additional critical data. | Thank you so much for your suggestion. Although the concept of this manuscript is very common, the information on white mugwort production, especially the nutrient management in organic farming, is still very few in Thailand and worldwide. Therefore, this data could be the basic information for both farmer and researcher for applying this data to practice in their field condition or research area. |

We are most grateful to you and the reviewers for helpful comments on the original version of our manuscript and consider our paper for publication in the journal. Follow the reviewer's comments we have revised the manuscript.

We deeply hope that this revised version of our manuscript in now suitable for publication.

Thank you very much indeed.

Sincerely yours,

Assist. Prof. Dr. ORNPRAPA THEPSILVISUT
Major of Agricultural Technology
Faculty of Science and Technology
Thammasat University
Klong Noeng, Klong Luang, Pathum Thani 12120
Tel: +66(0)2-564-4440 Ext 2356
Mobile: +66(0)96 995 3542
E-mail: ornprapa@hotmail.com, ornprapa@tu.ac.th
